# Investigation of the epidermal growth factor receptor mutation rate in non-small cell lung cancer patients and the analysis of associated risk factors using logistic regression

**DOI:** 10.3892/ol.2014.2160

**Published:** 2014-05-19

**Authors:** QINSHI PAN, YUMIN WANG, JIE CHEN, GANG XU, BICHENG CHEN, JINGYE PAN, KATE HUANG

**Affiliations:** 1 Department of Laboratory Medicine, The First Affiliated Hospital of Wenzhou Medical University, Wenzhou, Zhejiang, P.R. China; 2 Department of Intensive Care Unit, The First Affiliated Hospital of Wenzhou Medical University, Wenzhou, Zhejiang, P.R. China; 3 Department of Surgical Laboratory, The First Affiliated Hospital of Wenzhou Medical University, Wenzhou, Zhejiang, P.R. China; 4 Department of Pathology, The First Affiliated Hospital of Wenzhou Medical University, Wenzhou, Zhejiang, P.R. China

**Keywords:** mutation, non-small cell lung carcinoma, epidermal growth factor receptor, logistic regression analysis

## Abstract

The aim of the present study was to investigate the mutation rate of the epidermal growth factor receptor (EGFR) in non-small cell lung cancer (NSCLC) patients and to apply logistic regression analysis to investigate the factors associated with EGFR gene mutation to provide data for the treatment of NSCLC. Paraffin tissue, bronchoscopy or pleural effusion specimens were collected from 176 NSCLC patients following pathological diagnosis. The EGFR gene exon 19 delL747-S75linss and delL747-S752ins deletion mutations, and the exon 20 T790M and exon 21 L858R mutations were identified using amplification refractory mutation system analysis. The clinical data and laboratory results of the patients were collected, and the total mutation rate of the EGFR gene in exons 19, 20 and 21 in the 176 NSCLC patients was found to be 48.3% (85/176). In addition, the EGFR gene mutation rate in adenocarcinoma was found to be significantly higher than that in squamous cell and large cell carcinoma (χ^2^=12.454; P=0.002). Furthermore, the mutation rate was found to be significantly higher in females than in males (χ^2^=13.78; P=0.001). The rate of exon 19 mutation was 21.0% (37/176), whereas the rate of exon 20 T90M mutation was 1.7% (3/176) and that of exon 21 L858R mutation was 29.0% (51/176). The logistic regression analysis revealed that the female gender, adenocarcinoma, distant metastasis and chemotherapy are factors associated with EGFR gene mutation (P<0.05). The female gender resulted in an increased incidence (2.438 times that of males) of EGFR mutation. Similarly, adenocarcinoma, distant metastasis and chemotherapy exhibited an increase in EGFR mutation risk (by 2.571, 2.810 and 0.367 times, respectively). The rate of EGFR mutation was high in the NSCLC patients, predominantly in exons 21 and 19. Therefore, these factors (female gender, adenocarcinoma, distant metastasis and chemotherapy) may increase the probability of EGFR gene mutations.

## Introduction

Lung cancer-related mortality is the highest among all the cancer types, and its incidence is gradually increasing (1). Non-small cell lung cancer (NSCLC) is the most common type of lung cancer, accounting for 80% of all lung cancer cases, and includes squamous cell carcinoma, adenocarcinoma and large cell carcinoma. However, despite the continuous improvements in surgical resection, chemotherapy and radiation therapies, patients with lung cancer remain extremely vulnerable to relapse and mortality (2). The cure rate of lung cancer is extremely low and the average five-year survival rate of patients with lung cancer is <15% (3–6).

At present, the treatment of cancer depends predominantly on cytotoxic chemotherapy, however, the efficacy in the majority of solid tumors remains extremely limited and marked toxic side-effects have been identified, particularly in patients with lung cancer (4–6). The early detection of lung cancer is not easy and patients are often diagnosed in the middle or late stages of lung cancer and thus, the opportunity for surgical treatment is missed and the conventional drugs for chemotherapy exhibit limited effects. However, gefitinib is a drug that exhibits improved efficacy and safety in locally advanced or metastatic NSCLC patients. Furthermore, the epidermal growth factor receptor (EGFR) gene mutation is a predictor of gefitinib sensitivity in lung cancer (7,8). Thus, early detection of the EGFR mutation rate and its associated factors in lung tumors may present an important reference to individualize clinical treatment and improve treatment implementation in lung cancer, as well as to reduce the toxicity of drugs. EGFR gene mutations, including delE746-A750, delL747-p753inss, delL747-T75linss, delL747-S752ins, T790M and L858R, have been identified in NSCLC patients. However, various studies have found that the factors associated with the EGFR gene mutations in NSCLC patients are not entirely consistent, and therefore, a small number of studies have reported contradicting conclusions (9–11). In the present study, the EGFR gene exon 19 deletion, the exon 20 T790M mutation and the exon 21 L858R mutation were detected, and single factor logistic regression analysis was used to further analyze the factors associated with the EGFR mutation, to provide data for the treatment of NSCLC.

## Materials and methods

### Subjects

The specimens, including paraffin tissue, pleural effusion and bronchoscopy samples, were obtained from 176 NSCLC patients treated at the First Affiliated Hospital of Wenzhou Medical College (Wenzhou, Zhejiang, China) between January 2006 and February 2013. These cases were confirmed by histopathology and cytology. Of the 176 patients, 98 were male and 78 were female, aged between 33 and 89 years. The demographic characteristics of the 176 NSCLC patients are shown in Table I. The histopathological diagnosis of NSCLC was performed using the criteria of the 2004 World Health Organization/International Association for the Study of Lung Cancer lung cancer histological classification standards (12). This study was approved by the Institutional Ethics Review Board of the First Affiliated Hospital of Wenzhou Medical College and all patients provided written informed consent.

### Clinical data

The clinical data obtained from the patients included gender, age, pathological type, clinical stage, degree of differentiation, lymph node metastasis, distant metastasis, pleural effusion, family history of cancer, occupational exposure, chemotherapy, surgery, radiotherapy, tumor volume [tumor volume = (a × b^2^) / 2] and smoking index (number of cigarettes smoked per day × number of years).

### DNA extraction

DNA was extracted from the samples using a formalin-fixed, paraffin-embedded DNA extraction kit (Omega Corporation, Yarraville, Victoria, Australia) according to the manufacturer’s instructions. The DNA samples were examined for purity and concentration, and were diluted to a working concentration of 10 ng/μl.

### EGFR mutation analysis by the amplification refractory mutation system (ARMS)

ARMS analysis was conducted using a DxS EGFR mutation test kit (Amoy Diagnostics Co., Ltd, Xiamen, China), according to the manufacturer’s instructions.

### Statistical analysis

A comparison between the two groups was performed by the Mann-Whitney U test or Student’s t-test for measured variables. The differences between the rates of EGFR gene mutation were tested for statistical significance using χ^2^ or Fisher’s exact tests. The factors studied in patients with EGFR mutations were compared with the patients without EGFR mutations. Briefly, a univariate logistic regression analysis was conducted for each candidate variable and P<0.05 was considered to indicate a statistically significant difference. Next, a univariate logistic regression analysis was performed to identify which markers were associated with mutations of the EGFR gene (Table II). All data were analyzed using SPSS version 13.0 software (SPSS, Inc., Chicago, IL, USA).

## Results

### Mutation status of the EGFR gene in NSCLC patients

In the 176 NSCLC patients, the total mutation rate of exons 19, 20 and 21 in the EGFR gene was 48.3% (85/176). The adenocarcinoma EGFR gene mutation rate (77/139) was significantly higher than that of squamous cell carcinoma (8/36) and large cell carcinoma (0/1) (χ^2^=12.454; P=0.002). In addition, the mutation rate in females (51/78) was significantly higher than that in males (34/98) (χ^2^=13.78; P=0.001). The mutation rate of exon 19 was 21.0% (37/176; 19 males and 18 females; 33 adenocarcinoma and four squamous cell carcinoma cases). The exon 20 T790M mutation rate was 1.7% (3/176), which included two cases of simultaneous exon 19 deletions and one case of simultaneously occurring exon 21 L858R mutations (two males and one female; two cases of adenocarcinoma and one case of squamous cell carcinoma), while the exon 21 L858R mutation rate was 29.0% (51/176; 17 males and 34 females; 45 adenocarcinoma and six squamous cell carcinoma cases) (Table I.)

### Univariate logistic regression analysis of factors associated with EGFR mutation

According to the clinical data, which included gender, age, histological type, clinical stage, degree of differentiation, lymph node metastasis, distant metastasis, pleural effusion, family history of cancer, occupational exposure and chemotherapy, a logistic regression was performed to identify the risk factors associated with EGFR mutation. The χ^2^ test revealed that clinical stage (X3), degree of differentiation (X4), lymph node metastasis (X5), pleural effusion (X7), family history of cancer (X8), occupational exposure (X9), surgery (X11) and radiotherapy (X12) were not associated with an increased risk of EGFR mutation. However, gender (X1), pathological type (X2), distant metastasis (X6) and chemotherapy (X10) were identified as risk factors associated with EGFR mutation. In addition, a t-test revealed that age (X13) and tumor volume (X14) were not risk factors associated with EGFR mutation, whereas smoking index (X15) was identified as a risk factor (Tables I and III)

The associated single factors were used to perform non-conditional logistic regression analysis, and the results revealed that gender [X1; P=0.011; odds ratio (Exp) (B)=2.423; 95% confidence interval (CI) for Exp (B)=1.27–4.799], pathological type [X2; P=0.039; Exp (B)=0.388; 95% CI for Exp (B)=0.158–0.953], distant metastasis [X6; P=0.004; Exp (B)=2.798; 95% CI for Exp (B)=1.383–5.658] and chemotherapy [X10; P=0.006; Exp (B)=0.364; 95% CI for Exp (B)=0.176–0.752] are the predominant risk factors associated with EGFR mutation. In addition, multidimensional logistic regression analysis was applied to analyze the correlation between EGFR mutation and gender, pathological type, distant metastasis and chemotherapy. The results revealed that the female gender results in an increased incidence (2.438) of EGFR mutation. Similarly, adenocarcinoma, distant metastasis and chemotherapy showed an increase in EGFR mutation risk by 2.571, 2.810 and 0.367 times, respectively (Tables IV and V).

## Discussion

Previous studies have shown that >70% of NSCLC patients with EGFR mutations are sensitive to EGFR-tyrosine-kinase inhibitor (TKI) drugs, compared with only 10% in patients without EGFR mutations (13). EGFR mutations occur predominantly in exons 18–21 and it has also been observed that the EGFR-TKI drug class exhibits different clinical responses in patients with different EGFR mutation types. For example, an insertion mutation in exon 20 often exhibits resistance to gefitinib or erlotinib, whereas exon 18 mutations exhibit moderate sensitivity to these drugs (less than that of exon 19 or 21 mutations). Furthermore, the survival time of patients with exon 19 mutations is longer than that of patients with exon 21 mutations when receiving the same EGFR-TKI treatment (14–16). These findings indicate that it is important to adequately distinguish between EGFR mutation status and mutation types for the targeted therapy of NSCLC.

In addition, significant differences in the EGFR mutation rate in NSCLC patients have been identified between Asian and non-Asian populations with rates of 26–40% and 2–12%, respectively. Furthermore, almost all mutations detected in adenocarcinoma tissue (17–21), were in the tyrosine kinase domains of 19 and 21 of exon, which usually account for ~90% of EGFR mutations. These two sites of mutation were also found to significantly correlate with the efficacy of EGFR-TKI treatment. Therefore, the accurate detection of genetic changes plays a decisive role in the clinical treatment of the two sites.

The present study revealed that in 176 NSCLC patients, the total mutation rate of the EGFR gene exons 19, 20 and 21 was 48.3% (85/176). In addition, the adenocarcinoma EGFR gene mutation rate (77/139) was significantly higher than that in squamous cell (8/36) and large cell (0/1) carcinoma. The total EGFR gene mutation rate was marginally higher than that reported in previous studies, however, the majority of mutations were detected in adenocarcinoma, which is consistent with previously reported results (19). This study also revealed that the mutation rate in females (51/78) was significantly higher than that in males, consistent with the results reported by Toyooka *et al* (22). The reasons for this remain unclear, however, it may be associated with different lifestyles, smoking habits and endocrine factors.

Previous studies have shown that the factors associated with EGFR gene mutations in NSCLC patients are not entirely consistent, and a small number of studies have reported contradicting conclusions (9–11). In the current study, univariate analysis revealed that gender, pathological type, distant metastasis, chemotherapy and smoking index are factors associated with EGFR gene mutations. Furthermore, unconditional logistic regression analysis was used and revealed that female gender, adenocarcinoma, distant metastasis and chemotherapy are also factors associated with EGFR gene mutations. The results showed that females have an increased incidence (2.438 times that of males) of EGFR mutation. Similarly, adenocarcinoma, distant metastasis and chemotherapy were found to exhibit an increased risk of EGFR mutation by 2.571, 2.810 and 0.367 times, respectively.

In conclusion, the EGFR gene mutation rate is higher in patients with NSCLC, predominantly in exons 21 and 19. This study provides specifc data for the study of EGFR mutations in lung cancer research and treatment.

## Figures and Tables

**Table I:** Demographic characteristics of 176 NSCLC patients and χ^2^ results.

	Cases, n			
			
Project code (variable)	EGFR mutation^a^	EGFR non-mutation	χ^2^	P-value
X1 (gender)	83	93	13.78	0.001
Male	34	64		
Female	49	29		
X2 (pathological type)	83	93	12.454	0.002
Adenocarcinoma	75	64		
Squamous cell carcinoma	8	28		
Large cell carcinoma	0	1		
X3 (clinical stage)^b^	45	49	5.021	0.413
Ia	1	1		
Ib	1	3		
IIa	1	1		
IIb	0	0		
IIIa	6	14		
IIIb	5	6		
IV	31	24		
X4 (histological differentiation)^b^	54	46	1.494	0.684
Poorly-differentiated	34	31		
Poorly-to moderately-differentiated	4	3		
Moderately-differentiated	12	11		
Well-differentiated	4	1		
X5 (lymph node metastasis)^b^	82	93	0.090	0.765
Yes	44	52		
No	34	59		
X6 (distant metastasis)^b^	82	93	8.453	0.004
Yes	48	34		
No	34	59		
X7 (pleural effusion)^b^	82	93	0.362	0.547
Yes	29	37		
No	53	56		
X8 (family history of cancer)^b^	81	92	1.746	0.186
Yes	7	14		
No	74	78		
X9 (occupational exposure)	83	93	2.109	0.146
Yes	4	10		
No	79	83		
X10 (chemotherapy)	83	93	3.992	0.046
Yes	44	63		
No	39	30		
X11 (surgery)^b^	77	89	0.073	0.787
Yes	33	40		
No	44	49		
X12 (radiotherapy)^b^	82	89	2.891	0.089
Yes	8	3		
No	74	86		

a 19 delL747-S75linss or delL747-S752ins is 19 delL.

b Data not available for all patients.

NSCLC, non-small cell lung cancer.

**Table II:** Assignment values of the univariate logistic regression analysis.

Variables	Project code	Assignment value
Gender	X1	Female, 0; male, 1
Pathological type	X2	Adenocarcinoma, 1; squamous cell carcinoma, 2; large cell carcinoma, 3
Clinical stage	X3	Ia, 1; Ib, 2; IIa, 3; IIb, 4; IIIa, 5; IIIb, 6; IV, 7
Degree of differentiation	X4	Poorly-differentiated, 1; poorly- to moderately-differentiated, 2; moderately-differentiated, 3; well-differentiated, 4
Lymph node metastasis	X5	No, 0; yes, 1
Distant metastasis	X6	No, 0; yes, 1
Pleural effusion	X7	No, 0; yes, 1
Family history of cancer	X8	No, 0; yes, 1
Occupational exposure	X9	No, 0; yes, 1
Chemotherapy	X10	No, 0; yes, 1
Surgery	X11	No, 0; yes, 1
Radiotherapy	X12	No, 0; yes, 1
Age, years	X13	Specific data
Tumor volume	X14	Specific data
Smoking index	X15	Specific data
EGFR mutation	Y	No, 0; yes, 1

EGFR, epidermal growth factor receptor.

**Table III:** T-test/Mann-Whitney U test results for EGFR mutation and EGFR non-mutation in NSCLC patients.

		Cases, n			
				
Project code	Variable	EGFR mutation	EGFR non-mutation	Student’s t-test	P-value
X13	Age	83	93	−0.815^a^	0.416
X14	Tumor volume	20	22	145.500^b^	0.060
X15	Smoking index	83	93	2463.500^b^	0.001

a T-test; and

b Mann-Whitney U test.

EGFR, epidermal growth factor receptor; NSCLC, non-small cell lung cancer.

**Table IV:** Logistic analysis of variables associated with EGFR mutations from NSCLC patients.

Variable	B	Standard error	Wald	df	P-value	Exp (B)	95% CI for Exp (B)
Gender	0.885	0.349	6.438	1	0.011	2.423	1.23–4.799
Pathological type	−0.947	0.459	4.262	1	0.039	0.388	0.158–0.953
Distant metastasis	1.029	0.359	8.198	1	0.004	2.798	1.383–5.658
Chemotherapy	−1.011	0.370	7.458	1	0.006	0.364	0.176–0.752

EGFR, epidermal growth factor receptor; NSCLC, non-small cell lung cancer; df, degree of freedom; CI, confidence interval; Exp, odds ratio; Wald, Chi-square value = (B/standard error)^2^.

**Table V:** Logistic analysis of associated factors for EGFR mutations from NSCLC patients.

Variable	B	Standard error	Wald	df	P-value	Exp (B)	95% CI for Exp (B)
Female	0.891	0.349	6.527	1	0.011	2.438	1.231–4.831
Adenocarcinoma	0.944	0.469	4.055	1	0.044	2.571	1.026–6.446
Distant metastasis	1.033	0.359	8.273	1	0.004	2.810	1.390–5.682
Chemotherapy	−1.170	0.460	6.461	1	0.011	0.367	0.178–0.757

EGFR, epidermal growth factor receptor; NSCLC, non-small cell lung cancer; df, degree of freedom; CI, confidence interval; Exp, odds ratio; Wald, Chi-square value = (B/standard error)^2^.
